# Drug sequestration and metabolite formation: key pharmacokinetic challenges for levosimendan use during ECMO support in cardiogenic shock

**DOI:** 10.1186/s40560-026-00884-5

**Published:** 2026-04-24

**Authors:** Nicolas Tebib, Lucas Liaudet, Zied Ltaief

**Affiliations:** Service of Adut Intensive Care Medicine, University Hospital and Faculty of Biology and Medicine, Rue du Bugnon 46, 1011 Lausanne, Switzerland

**Keywords:** Levosimendan, Extracorporeal membrane oxygenation, ECMO, Pharmacokinetics, Cardiogenic shock

## Abstract

**Background:**

In patients with refractory cardiogenic shock, veno-arterial extracorporeal membrane oxygenation (ECMO) provides temporary circulatory support, but optimal strategies to promote myocardial recovery and facilitate ECMO weaning remain uncertain. Levosimendan is a calcium-sensitizing inotrope and vasodilator that improves cardiac performance and may support cardiac function and shorten the duration of ECMO support.

**Discussion:**

The recent multicenter randomized placebo-controlled LEVOECMO trial demonstrated no benefit of early levosimendan infusion on ECMO weaning or clinical outcomes, providing robust evidence against routine and early use. Several pharmacokinetic factors may have attenuated levosimendan efficacy in this setting. Levosimendan is prone to sequestration within the ECMO circuit, particularly early after cannulation. Moreover, its short half life and its reliance on hepatic and intestinal metabolism for the generation of the long-acting active metabolite may limit its effectiveness during the early phase of cardiogenic shock, when high incidence of hepatic dysfunction and mesenteric hypoperfusion may contribute to reduced active metabolite production. In addition, renal replacement therapy is frequently required in ECMO patients and may further reduce plasma concentrations of active metabolites. Taken together, and in the absence of therapeutic drug monitoring, these factors may result in highly variable and unpredictable exposure to active metabolites among patients. These considerations raise the hypothesis that alternative strategies such as delayed initiation with therapeutic drug monitoring might improve exposure to levosimendan and its active metabolites.

**Conclusion:**

While recent evidence discourages routine early levosimendan use during ECMO for cardiogenic shock, a personalized, pharmacology-driven approach needs further investigation before a definitive conclusion.

**To the Editor,**

Cardiogenic shock is among the most critical conditions encountered in intensive care. Despite significant advances in monitoring and supportive therapies, mortality rates remain high [1]. For selected patients with refractory cardiogenic shock unresponsive to conventional therapy, veno-arterial extracorporeal membrane oxygenation (ECMO) remains the only temporary circulatory support system that provides immediate circulatory and respiratory assistance [2]. ECMO functions as a bridge to decision, heart recovery, transition to other durable mechanical support or transplantation. During prolonged ECMO support, the exposure of blood components to the circuit and oxygenation membrane, as well as the requirement for large vascular access and anticoagulation, increases the risk of infectious, thrombotic, and hemorrhagic complications [3]. Therefore, effective management of patients receiving ECMO requires daily assessment of myocardial recovery to balance adequate circulatory support with the earliest safe weaning.

In this setting, pharmacological strategies based on the levosimendan administration remain an attractive solution. Levosimendan exerts its inotropic effect by increasing the sensitivity of cardiac contractile proteins to calcium, thereby enhancing myocardial contractility without increasing intracellular calcium concentration, resulting in improved cardiac performance without impairing relaxation or increasing myocardial oxygen consumption. In addition, levosimendan exerts a vasodilatory effect on peripheral circulation and coronary vessels, decreasing cardiac afterload and improving coronary blood flow. This makes levosimendan theoretically attractive in the context of heart recovery and ECMO weaning [4–6].

However, the results from studies across different critical care populations have been heterogenous and the role of levosimendan in this context remains debated. In a recent meta-analysis including 2083 patients across 16 studies, levosimendan was associated with improved ECMO weaning and reduced mortality. The authors concluded that, despite this signal, further high-quality studies were required to confirm these findings. Importantly, all included studies were single-center, retrospective, with small sample sizes and heterogeneous criteria for weaning resulting in a high risk of bias and limited generalizability [7].

More recently, Combes et al. published the first multicentric randomized placebo-controlled trial evaluating early levosimendan infusion in patients with severe cardiogenic shock supported with ECMO. The study compared a 24-h infusion of levosimendan (0.2 µg/kg/min), started within 48 h after cannulation, versus placebo, with successful ECMO weaning as the primary endpoint (the LEVOECMO trial) [8]. The LEVOECMO trial found no significant difference in the rate of successful ECMO weaning, nor any improvement in secondary outcomes, including mortality and duration of ECMO support. This well-designed study provides robust, high-level evidence regarding the early use of levosimendan for ECMO weaning in cardiogenic shock. Still, we believe that some pharmacokinetic issues need to be considered in the interpretation of these results, as rightly mentioned by the authors in the limitation section of their article [8]. From these considerations, we suggest the hypothesis that patients treated with levosimendan may have experienced subtherapeutic drug exposure, which could have contributed to the negative trial results.

First, due to its lipophilic nature, high protein binding and ionization degree at physiological pH, levosimendan is prone to sequestration in the ECMO circuit, which could result in reduced pharmacological effects. Indeed, up to 33% levosimendan sequestration has been reported in an ex vivo ECMO study [9], while a clinical study in the pediatric population reported that up to 78% levosimendan was lost by sequestration during ECMO support [10]. Such sequestration could be of particular concern with a newly inserted ECMO circuit in which drug adsorption is likely to be maximal, resulting in potential underexposure to levosimendan [11], especially at the dosing strategy used in the LEVOECMO trial: a loading dose was not applied (which is frequently chosen to avoid hypotension) and the infusion rate was based on previous studies in heart failure patients not supported by ECMO [12, 13].

Second, levosimendan has a very short elimination half-life (t ½: 1–1.5 h) due to extensive (95%) metabolism into inactive metabolites. About 5% of levosimendan is secreted into the bile and transformed by the gut microbiota into an intermediate metabolite: OR-1855, which is further metabolized by liver acetylation into the active metabolite: OR-1896. OR-1855 and OR-1896 exhibit long half-lives (t ½: ~ 70–80 h), and OR-1896 is responsible for the long-lasting inotropic actions of levosimendan [10]. Liver dysfunction and splanchnic hypoperfusion are common during the early phase of cardiogenic shock [14–17]. Furthermore, experimental data showed that VA-ECMO impairs intestinal and liver microcirculatory blood flow, despite improvement of the macrocirculation [18, 19]. Theoretically, such alterations could reduce the generation of levosimendan metabolites, limiting its long-term inotropic actions. Meaningful pharmacokinetic data obtained in cirrhotic patients with moderate liver dysfunction showed, indeed, decreased peak concentration and prolonged elimination half-life of OR-1855 and OR-1896 [20], consistent with less efficient and slower metabolism of levosimendan in conditions of liver impairment. It is also noteworthy that peripheral VA-ECMO is frequently associated with lower body hyperoxia, which may expose the intestine to markedly elevated oxygen pressure [21, 22]. Intestinal hyperoxia is an important driver of gut dysbiosis [23, 24], with expansion of oxygen-tolerant bacteria at the expense of commensal anaerobes [21], which could impair microbial-dependent metabolism of levosimendan. In support of this hypothesis, the formation of the metabolites OR-1855 and OR-1896 is extremely reduced in neonates and infants with an immature intestinal microbiota [25], composed primarily of aerotolerant microorganisms [23].

Third, nearly 80% of patients develop acute kidney injury and half require renal replacement therapy (RRT) in the context of cardiogenic shock and ECMO [26]. In the LEVOECMO trial, the incidence of renal replacement therapy (RRT) in the levosimendan arm of the study reached 28.7% during the 30-day follow-up [8]. Although renal failure has been shown to prolong the half-life of OR-1855 and OR-1896, both metabolites are dialyzable (while levosimendan is not), and their clearance increases approximately tenfold during hemodialysis sessions [27]. In addition, pharmacokinetic modeling by Bertin et al. identified continuous RRT as an independent covariate associated with increased elimination of levosimendan metabolites [25]. Therefore, RRT, most especially in the continuous mode, increases the elimination of levosimendan metabolites, which may have attenuated their sustained pharmacological actions in VA-ECMO patients undergoing RRT in the LEVOECMO trial (Fig. 1).

**Fig. 1:**
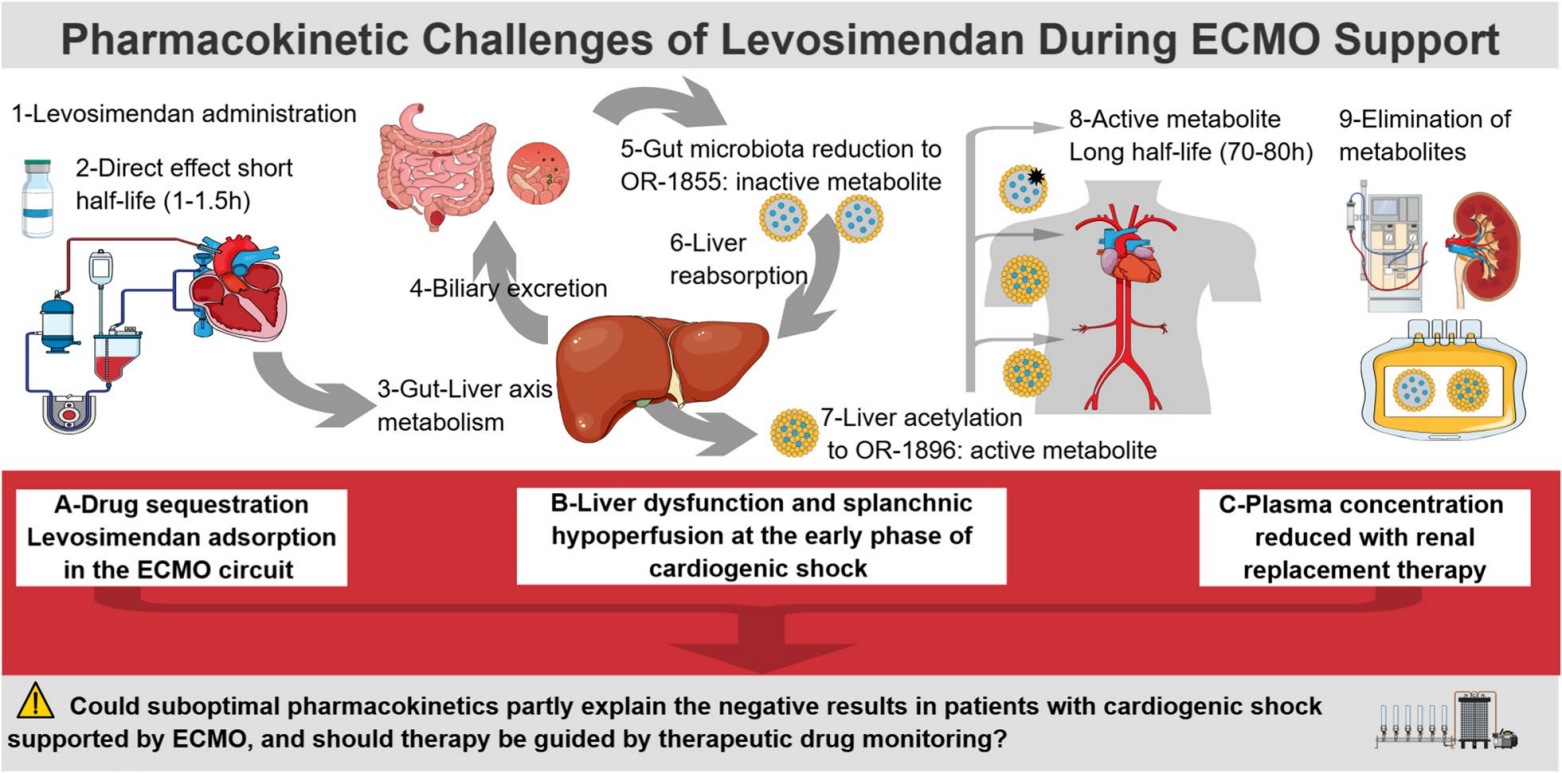
Pharmacokinetic challenges of levosimendan use during ECMO support in cardiogenic shock. After intravenous administration (**1**), levosimendan exerts a direct hemodynamic effect, but it has a short elimination half-life (1–1.5 h) (**2**). A fraction of levosimendan undergoes gut–liver axis metabolism (**3**) and is excreted into bile (**4**). In the intestinal lumen, levosimendan is reduced by the gut microbiota to the intermediate inactive metabolite OR-1855 (**5**), which is subsequently reabsorbed (**6**) and acetylated in the liver to form the active metabolite OR-1896 (**7**). OR-1896 and OR-1855 exhibit long elimination half lives (~ 70–80 h), and only OR-1896 accounts for the prolonged inotropic effects of levosimendan (**8**). Both metabolites are eliminated predominantly by renal excretion and are dialyzable (**9**). During ECMO support, three major pharmacokinetic disturbances may reduce effective exposure to levosimendan and its active metabolite: A: adsorption of levosimendan in the ECMO circuit, B: impaired generation of active metabolites related to liver dysfunction and splanchnic hypoperfusion at the early phase of cardiogenic shock, and C: enhanced clearance of OR-1855 and OR-1896 during renal replacement therapy. These mechanisms may contribute to insufficient active drug concentrations and attenuated pharmacodynamic response. *: OR-1855 has no hemodynamic effect

Based on these considerations, one may question whether alternative strategies of levosimendan treatment in patients supported by VA-ECMO might have yielded different clinical effects. For instance, delaying administration until partial recovery of liver and splanchnic perfusion could improve intestinal and hepatic-dependent formation of the active metabolites, thereby enhancing their sustained inotropic effects. Also, administering levosimendan after a time interval following ECMO cannulation could reduce early circuit-related adsorption. Finally, assessing plasma levels of levosimendan and its metabolites through therapeutic drug monitoring might permit dosing adjustments to achieve desired pharmacological effects. However, due to the lack of widely available clinical assay and validated pharmacodynamic target concentrations, such monitoring should be viewed as a research tool but cannot be currently applied as a practical clinical strategy.

As a last comment, we also believe that the negative results of the trial do not necessarily exclude any positive biological effects of levosimendan on cardiac function. Although the primary endpoint of successful VA-ECMO weaning is clinically meaningful, it may not represent the ideal pharmacodynamic endpoint for levosimendan. ECMO weaning requires a tailored approach balancing a complex composite of myocardial recovery, end-organ function and clinician-driven weaning decisions. Accordingly, such endpoint may dilute some specific effects of levosimendan and its metabolites on myocardial performance*.*

In conclusion, while the recent evidence discourages routine early use of levosimendan during ECMO [8], a more nuanced, patient-specific and pharmacology-based approach deserves further investigation before definitively abandoning this drug in all patients supported with extracorporeal circulation.

## Data Availability

No datasets were generated or analyzed during the current study.

## Abbreviations

ECMO: Extracorporeal membrane oxygenation
LEVOECMO: Levosimendan to facilitate weaning from ECMO in patients with severe cardiogenic shock. The LEVOECMO randomized clinical trial
t½: Elimination half-life
OR-1855: Intermediate metabolite of levosimendan
OR-1896: Active metabolite of levosimendan
